# Intensive Care Management of Anti-Mi2–Positive Dermatomyositis: Lupus Overlap Syndrome

**DOI:** 10.7759/cureus.104509

**Published:** 2026-03-01

**Authors:** Maria Dioselina Ruiz Barrera, Maria Alaciel Galvan Merlos, Jorge Omar Castro Meza, Dafne Thamara Ayala Davila, Janeet Garduño Becerra

**Affiliations:** 1 Internal Medicine, Hospital Regional de Alta Especialidad de Ixtapaluca, Ixtapaluca, MEX; 2 Critical Care Medicine, Hospital Regional de Alta Especialidad de Ixtapaluca, Ixtapaluca, MEX; 3 Pathology and Laboratory Medicine, Hospital Regional de Alta Especialidad de Ixtapaluca, Ixtapaluca, MEX

**Keywords:** adult-onset dermatomyositis, anti-mi-2a antibodies, auto immune, autoimmune overlap syndrome, critical care and internal medicine education, hospital mexico, serum creatinine phosphokinase, sle and myositis overlap syndrome, sytemic lupus erythematosus, therapeutic plasmapheresis

## Abstract

We report the case of a 49-year-old woman diagnosed with an autoimmune overlap syndrome characterized by anti-Mi2-positive dermatomyositis and systemic lupus erythematosus (SLE), presenting with extreme hyper-creatine kinase (CK) levels (>3000 U/L), progressive muscle weakness, and respiratory failure requiring intensive care management. Muscle biopsy revealed chronic inflammatory myopathy with perifascicular necrosis, and immunologic testing showed antinuclear antibody (ANA) 1:640, anti-double-stranded (ds)DNA, and anti-Mi2 positivity, confirming the diagnosis.

During her clinical course, she developed hypoxemic respiratory failure, multiple endocrine dysfunctions (central hypopituitarism and autoimmune hypothyroidism), and nosocomial infections, all of which were successfully controlled. She received intravenous immunoglobulin (IVIG) therapy followed by five sessions of therapeutic plasma exchange (PLEX) (1.5 plasma volumes replaced with albumin), achieving partial muscle strength recovery, a 60% reduction in CK levels, and hemodynamic stabilization.

This case represents a rare and severe form of dermatomyositis-lupus overlap syndrome with critical evolution and favorable response to combined immunomodulatory therapy. It highlights the importance of early diagnosis, multidisciplinary management, and the timely use of therapeutic PLEX as a rescue treatment for refractory autoimmune diseases in critically ill patients.

## Introduction

Idiopathic inflammatory myopathies (IIMs) comprise a heterogeneous spectrum of systemic autoimmune diseases characterized by proximal muscle weakness, elevation of muscle enzymes, and histopathologic findings of muscle necrosis and inflammation [1,2]. Within this group, dermatomyositis represents the phenotype with the greatest cutaneous specificity, including heliotrope rash and Gottron papules, and is characterized by complement-mediated microangiopathy [1,3]. Dermatomyositis is also associated with a well-defined serologic profile of myositis-specific autoantibodies (MSA). Among these, anti-Mi2 antibodies are typically associated with a more favorable clinical course, better response to glucocorticoids and intravenous immunoglobulin (IVIG), and lower mortality compared with other autoantibody profiles such as anti-MDA5 or anti-TIF1-γ [3]. Nevertheless, a minority of anti-Mi2-positive patients may develop extreme hyper-creatine kinase (CK) levels, respiratory involvement, severe dysphagia, and organ failure, requiring admission to the intensive care unit [4,5]. Dermatomyositis-systemic lupus erythematosus (SLE) overlap syndrome is uncommon but represents a clinically challenging entity. [5]

This overlap combines inflammatory muscle and cutaneous involvement with systemic immunologic features, including positive antinuclear antibodies (ANA_, anti-double-stranded (ds)DNA antibodies, and hypocomplementemia [6]. Patients with overlap syndromes often exhibit a higher burden of comorbidities, such as autoimmune liver disease, cytopenias, and serositis [5]. Additionally, atypical neuromuscular and endocrine manifestations may be present, frequently delaying diagnosis and initiation of appropriate therapy [6,7]. Idiopathic inflammatory myopathies are clinically and biologically heterogeneous disorders, with wide variability in disease severity, extramuscular involvement, and response to immunosuppressive therapy, which complicates both diagnosis and therapeutic decision-making, particularly in severe or atypical presentations [8].

From a pathophysiological perspective, dermatomyositis is characterized by immune-mediated microangiopathy, complement activation, and perifascicular muscle fiber injury, with distinct clinical phenotypes determined by myositis-specific autoantibodies. Anti-Mi2-positive dermatomyositis is classically associated with prominent cutaneous manifestations and a more favorable prognosis; however, severe muscular involvement with marked hyper-CK levels and systemic complications has been increasingly recognized [9].

Overlap syndromes involving dermatomyositis and other systemic autoimmune diseases, particularly SLE, represent a distinct and challenging subset, often associated with higher disease burden, multisystem involvement, and diagnostic delay due to overlapping clinical and immunologic features [8]. Respiratory involvement in inflammatory myopathies, whether secondary to respiratory muscle weakness or associated pulmonary disease, constitutes a major determinant of morbidity and mortality and frequently necessitates intensive care management in acute or rapidly progressive cases [5].

In this context, severe neurogenic dysphagia and respiratory failure secondary to diaphragmatic and respiratory muscle weakness markedly increase morbidity and mortality [5]. These complications necessitate rapid and coordinated therapeutic decisions, including ventilatory support, definitive enteral nutrition, and aggressive control of systemic inflammation [5,10].

Glucocorticoids and steroid-sparing immunosuppressive agents, such as methotrexate, azathioprine, and mycophenolate mofetil, constitute the cornerstone of treatment for inflammatory myopathies [1,2]. However, a subset of patients demonstrates refractoriness or acute clinical deterioration despite standard immunosuppressive therapy [2,7]. In such cases, sequential immunomodulatory strategies using IVIG followed by therapeutic plasma exchange (PLEX) may be lifesaving. These therapies act by reducing circulating autoantibodies, immune complexes, and proinflammatory mediators [1,11].

Although evidence is primarily derived from small series and case reports, early implementation of these strategies has been associated with improvement in muscle strength, reduction in CK levels, and hemodynamic stabilization [11]. Optimal outcomes are more likely when treatment is delivered within a multidisciplinary framework involving intensive care, rheumatology, neurology, rehabilitation, nutrition, and speech therapy [10]. The present case is noteworthy due to the rarity of anti-Mi2-positive dermatomyositis-SLE overlap associated with extreme hyper-CKemia requiring intensive care management [3,5].

It further highlights a severe multisystem presentation involving neuromuscular, respiratory, endocrine, and hepatic domains [12,13]. The favorable clinical response observed following IVIG therapy and five sessions of therapeutic PLEX underscores the potential role of combined immunomodulatory therapy in refractory cases [1,14]. Finally, this case illustrates the diagnostic challenges, including overlap with polyneuropathies, concomitant infections, and multifactorial coagulopathy, as well as the importance of time-critical decision-making in the ICU setting [5,10].

## Case presentation

A 49-year-old woman with no prior diagnosis of autoimmune, neuromuscular, or chronic degenerative disease was admitted to our tertiary care center with a rapidly progressive neuromuscular syndrome complicated by respiratory failure. Her medical history was notable only for prolonged exposure to biomass smoke, estimated at approximately eight hours daily for more than four decades, a factor associated with chronic systemic inflammation and immune dysregulation. There was no family history of autoimmune disease, neuromuscular disorders, or malignancy.

The clinical course began three weeks before hospital admission with insidious-onset, symmetric proximal muscle weakness predominantly involving the pelvic girdle. The patient reported progressive difficulty rising from a seated position, climbing stairs, and ambulating independently, accompanied by early fatigability and myalgia. There was no documented evidence of darkened or cola-colored urine suggestive of clinically overt myoglobinuria during the initial phase of the illness. Over the following days, weakness extended to the upper extremities, significantly impairing activities of daily living.

Approximately 10 days after symptom onset, the patient developed progressive dysphagia, initially to solid foods and later to liquids, accompanied by persistent sialorrhea and choking episodes, suggesting bulbar muscle involvement and raising concern for impending respiratory compromise. No clear acute trigger for the current episode was identified. The patient denied any recent infections, febrile illness, vaccination, exposure to new medications, or toxic ingestion prior to symptom onset. There was no history of recent respiratory or gastrointestinal illness, and initial laboratory and microbiologic studies did not support an active infectious process. Although prolonged biomass smoke exposure is noted in her history, it represents a chronic environmental factor rather than an identifiable acute precipitant. Therefore, the presentation was considered idiopathic in onset, likely reflecting intrinsic autoimmune activation in the setting of evolving overlap connective tissue disease.

Several days later, the patient experienced exertional dyspnea that progressed to an episode of acute hypoxemia associated with transient loss of consciousness lasting approximately three minutes, followed by spontaneous recovery. She was evaluated at a regional hospital, where an acute inflammatory neuropathy was suspected based on the ascending pattern of weakness and cerebrospinal fluid findings. Consequently, she was referred to our institution for advanced diagnostic evaluation and management.

Initial physical examination and neurological assessment

On admission, the patient was alert, oriented, and hemodynamically stable. Vital signs were within normal limits, and oxygen saturation was preserved at rest. Dermatologic examination revealed a violaceous periorbital discoloration consistent with a heliotrope rash. No Gottron’s papules, shawl sign, V-sign, mechanic’s hands, digital ulcers, calcinosis, or other cutaneous manifestations of dermatomyositis were observed. Photographic documentation of the cutaneous finding was not available. Neurological examination demonstrated severe symmetric weakness involving both proximal and distal muscle groups of the upper and lower extremities, graded as 2/5 on the Medical Research Council scale. Muscle tone was diffusely reduced, and deep tendon reflexes were markedly diminished. Sensory examination was unremarkable, and no cranial nerve deficits were initially identified. There were no signs of meningeal irritation. Cardiopulmonary examination did not reveal abnormalities at presentation. However, given the rapid progression of neuromuscular symptoms and bulbar involvement, the patient was closely monitored for respiratory deterioration.

Laboratory and immunologic evaluation

Comprehensive laboratory evaluation revealed hematologic abnormalities, marked elevation of muscle enzymes, autoimmune serologic activity, endocrine dysfunction, and coagulation abnormalities consistent with a severe multisystem autoimmune inflammatory process (Table 1).

**Table 1:** Key laboratory findings in the patient with anti-Mi2–positive dermatomyositis-lupus overlap Comprehensive laboratory profile demonstrated severe hyper-CKemia, normocytic anemia, lymphopenia, coagulation abnormalities, cholestatic liver enzyme elevation, endocrine dysfunction, and autoimmune serologic activity consistent with anti-Mi2-positive dermatomyositis-SLE overlap. CK: Creatine kinase; SLE: Systemic lupus erythematosus; PT: Prothrombin time; INR: International normalized ratio; aPTT: Activated partial thromboplastin time; TSH: Thyroid-stimulating hormone; CPK: Creatine phosphokinase; CPK-MB: Creatine phosphokinase myocardial band; ANA: Antinuclear antibodies; dsDNA: Double-stranded DNA; SSA: Sjögren's-syndrome-related antigen A

Test	Parameter	Result	Units	Reference range
Complete blood count (CBC)	Red blood cells (RBC)	3.3	x10⁶/µL	4.2-5.4
	Hemoglobin	10	g/dL	12-16
	Hematocrit	29	%	36-48
	Mean corpuscular volume (MCV)	87.9	fL	80-96
	Mean corpuscular hemoglobin (MCH)	30.3	pg	27-33
	Mean corpuscular hemoglobin concentration (MCHC)	34.5	g/dL	32-36
	Red cell distribution width (RDW)	17.3	%	11.5-14.5
	White blood cells (WBC)	6	x10³/µL	4.5-11.0
	Neutrophils	5.57	x10³/µL	2.0-7.5
	Lymphocytes	0.27	x10³/µL	1.0-4.0
	Monocytes	0.19	x10³/µL	0.2-0.8
	Eosinophils	0	x10³/µL	0.0-0.5
	Basophils	0.01	x10³/µL	0.0-0.2
	Platelets	178	x10³/µL	150-450
	Mean platelet volume	8.7	fL	7.5-11.5
Coagulation and basic metabolic panel	Prothrombin time (PT)	18.8	sec	11-13.5 sec
	PT (%)	52	%	>70%
	International normalized ratio (INR)	1.69	—	0.8-1.2
	Activated partial thromboplastin time (aPTT), partial thromboplastin time, activated with kaolin (TTPA)	72.9	sec	25-35 sec
	Blood urea nitrogen (BUN)	4	mg/dL	7-20
	Creatinine	0.12	mg/dL	0.6-1.2
	Urea	9	mg/dL	10-50
	Sodium	138	mmol/L	135-145
	Potassium	2.6	mmol/L	3.5-5.0
	Chloride	105	mmol/L	98-107
	Calcium	8	mg/dL	8.5-10.5
	Phosphorus	2.9	mg/dL	2.5-4.5
	Magnesium	2.23	mg/dL	1.7-2.2
	Glucose	98	mg/dL	70-100
	Uric acid	2	mg/dL	2.6-6.0
	Albumin	3.9	g/dL	3.5-5.0
Liver function panel and enzymes	Alkaline phosphatase (ALP)	475	U/L	44-147
	Aspartate aminotransferase (AST)	43	U/L	10-40
	Alanine aminotransferase (ALT)	24	U/L	7-56
	Total bilirubin (BT)	0.53	mg/dL	0.1-1.2
	Direct bilirubin (BD)	0.42	mg/dL	0-0.3
	Indirect bilirubin (BI)	0.11	mg/dL	<1.0
	Total proteins	6.1	g/dL	6.0-8.3
	Albumin	1.9	g/dL	3.5-5.0
	Globulins	4.2	g/dL	2.0-3.5
	Gamma-glutamyl transferase (GGT)	312	U/L	9-48
Heavy metals and toxicology	Blood mercury	0.7	µg/L	<10
	Blood lead	3.34	µg/dL	<5
Hormonal profile	Adrenocorticotropic hormone (ACTH)	12	pg/mL	7.2-63
	Morning cortisol	7.7	µg/dL	5-25
	Growth hormone	3.22	ng/mL	0.01-5.0
	Prolactin	8.3	ng/mL	<20
	IGF-1 (somatomedin C)	53	ng/mL	117-329
	Estradiol (serum)	10	pg/mL	10-60 (premenopausal female)
	Follicle-stimulating hormone (FSH)	0.75	IU/L	3-10 (follicular phase)
	Luteinizing hormone (LH)	0.5	IU/L	2-15 (follicular phase)
	Progesterone	0.14	ng/mL	0.1-0.3 (follicular phase)
Thyroid function panel	Total triiodothyronine (T3)	20	ng/dL	80-200
	Total thyroxine (T4)	0.91	µg/dL	5.0-12.0
	Thyroid-stimulating hormone (TSH)	7.83	µIU/mL	0.5-4.5
	Free T3	1.14	pg/mL	2.3-4.2
	Free T4	0.56	ng/dL	0.93-1.7
Enzymatic curve: Creatine phosphokinase (CPK) and CPK-myocardial band (MB)	CPK	3079	U/L	26-192
	CPK-MB	480.4	U/L	0-25
Autoimmune panel	Antinuclear antibodies (ANA)	0.26388889	Granular nuclear pattern	Negative or <1:80
	ANA	0.48611111	Granular nuclear pattern	↑
	Anti-double-stranded (ds)DNA	35.5	IU/mL	Positive (>25)
	Anti-dsDNA	21	IU/mL	Borderline
	Anti-dsDNA	22.5	IU/mL	Borderline
	Anti-Sjögren's-syndrome-related antigen A (SSA) (Ro)	107.9	CU	Negative <20
	Anti-Sjögren's-syndrome-related antigen B (SSB) (La)	3.3	CU	Negative <20
	Anti-acetylcholine (ACh) receptor (modulating)	8%	%	Negative <15%
	Anti-tiroglobulin	7.89	IU/mL	Negative <4
	Anti-thyroid peroxidase (TPO)	1	IU/mL	Negative <5
	Anti-Mi2α/Mi2β	Detected	—	Not detectable (↑)
	Anti-striated muscle antibody	Negative	—	Negative
	Anti-smooth muscle antibody (SMA)	01:40	Titer	Negative <1:40 (Borderline)
	Anti-SMA	0.097	Titer	Negative
	Anti-liver-kidney microsomal (LKM)	0.25	Ratio	<0.5 (Negative)
	Antineutrophil cytoplasmic antibody (ANCA) (perinuclear (P)-ANCA)	3.2	UC	Negative <20
	ANCA (cytoplasmic (C)-ANCA)	9.5	UC	Negative <20
	IgA	565	mg/dL	70-400 (↑)
	IgG	3853	mg/dL	700-1600 (↑↑)
	IgM	148	mg/dL	40-230
	IgE	527	UI/mL	<100 (↑↑)
	Aldolase	17	U/L	<7.6 (↑↑)
	Aldolase	9.4	U/L	↑
Urinalysis and 24-hour urine tests	24-hour urine volume	2.5	L/24h	1-2
	24-hour urine volume	350	mL/24h	800-2000 (↓)
	Creatinine (urinary)	0.29	g/24h	0.5-2
	Microalbuminuria	27.7	mg/24h	<30
	Proteinuria (total)	872	mg/24h	<150 (↑↑)
	Proteinuria	371.88	mg/24h	<150 (↑)
CSF analysis	Erythrocytes	5,000	cells/µL	0
	Leukocytes	9	cells/µL	0-5 (↑)
	Neutrophils	60	%	0%-6% (↑↑)
	Lymphocytes	40	%	>60% (↓)
	Glucose	43	mg/dL	50-80 (↓)
	Proteins	115.8	mg/dL	15-45 (↑↑)
	Chloride	119	mmol/L	115-130
	Lactate dehydrogenase (LDH)	52	U/L	<40 (↑)

Hematologic analysis revealed normocytic normochromic anemia and marked lymphopenia, a pattern frequently observed in active SLE and severe autoimmune inflammation. Coagulation studies demonstrated prolongation of both PT and aPTT, suggesting mixed pathway involvement. In the critical care setting, this finding may reflect multifactorial processes, including inflammation-associated consumption, hepatic dysfunction, vitamin K deficiency, or disseminated intravascular coagulation. Although autoimmune-mediated coagulopathy was considered in the differential diagnosis, no antiphospholipid antibody testing was available to support a specific lupus-related mechanism.

**Figure 1:**
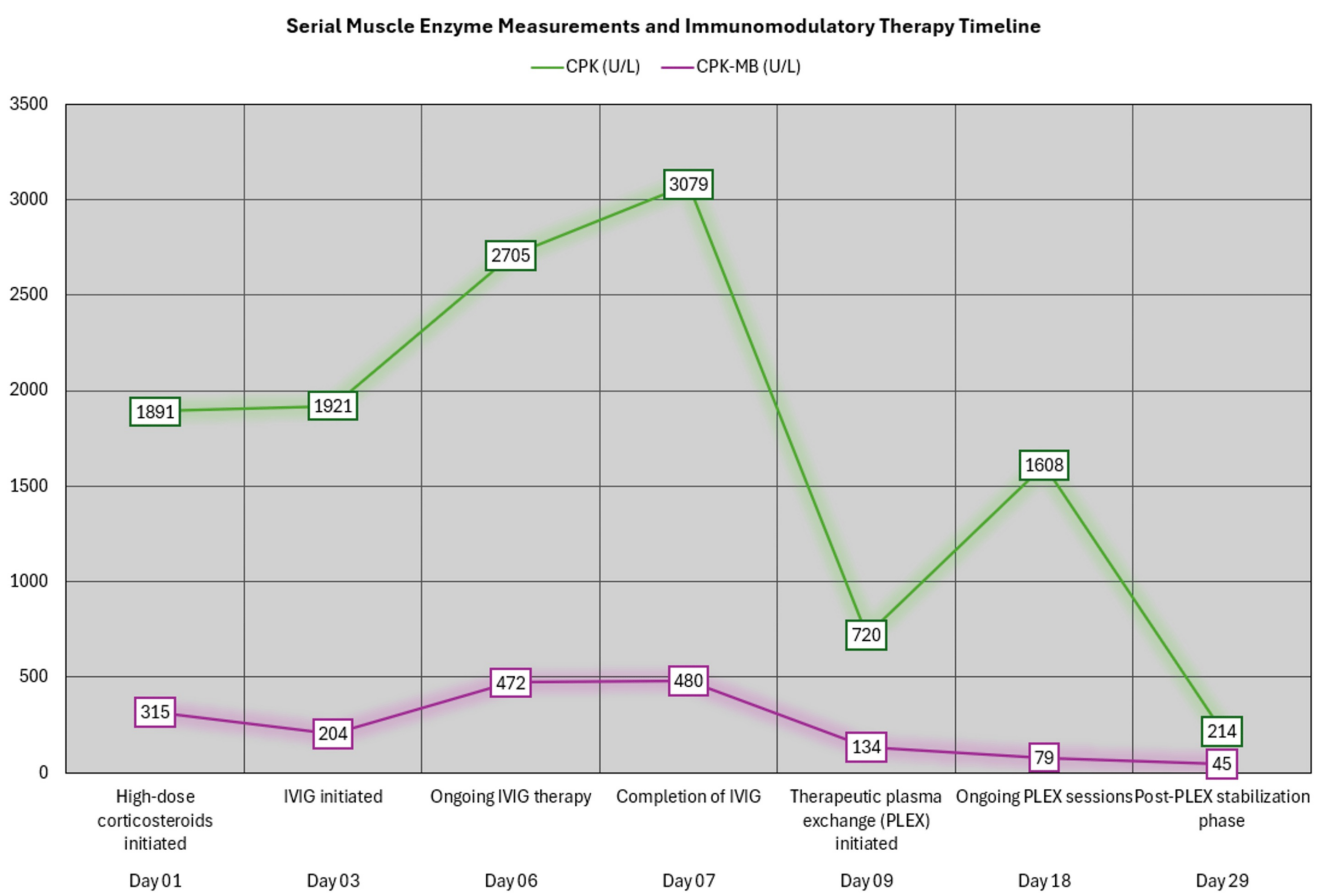
Serial muscle enzyme measurements and immunomodulatory therapy timeline This graph illustrates the temporal evolution of serum CPK (green) and CPK-MB (purple) levels across 29 days of hospitalization in relation to the sequential immunomodulatory therapies administered. The two curves are expressed in U/L. On day 1, coinciding with the initiation of high-dose corticosteroids, CPK was elevated at 1891 U/L and CPK-MB at 315 U/L. By day 3, when intravenous immunoglobulin (IVIG) was initiated, CPK remained elevated (1921 U/L) with a transient reduction in CPK-MB (204 U/L). During ongoing IVIG therapy (days 6–7), enzyme levels peaked, with CPK reaching 3079 U/L and CPK-MB 480 U/L, reflecting ongoing active myofiber necrosis. Following completion of IVIG and initiation of therapeutic plasma exchange (PLEX) on day 9, a marked decline in both markers was observed (CPK 720 U/L; CPK-MB 134 U/L), suggesting a biochemical response to therapy. A secondary transient rise in CPK was noted on day 18 (1608 U/L) during ongoing PLEX sessions, while CPK-MB continued to decline. By day 29, during the post-PLEX stabilization phase, both enzymes demonstrated significant reduction (CPK 214 U/L; CPK-MB 45 U/L), approaching near-normal levels. Overall, the graph demonstrates an initial severe hyper-CKemia with peak elevation during early disease activity, followed by progressive biochemical improvement temporally associated with combined corticosteroid therapy, IVIG, and PLEX. CPK: Creatine phosphokinase, CPK-MB: Creatine phosphokinase myocardial band, CK: Creatine kinase, IVIG: Intravenous immunoglobulin, PLEX: Plasma exchange

Biochemical analysis revealed significant electrolyte disturbances, including severe hypokalemia and mild hypocalcemia, both of which may exacerbate neuromuscular weakness. Renal function parameters remained preserved. Liver biochemistry showed a cholestatic-predominant pattern with markedly elevated alkaline phosphatase and gamma-glutamyl transferase, mild transaminase elevation, and hypoalbuminemia. Given the markedly elevated CK levels (>3,000 U/L), transaminase elevation cannot be definitively attributed to primary hepatic injury, as AST and ALT may also be released from skeletal muscle during active myositis. Although immune-mediated hepatic involvement was considered in the differential diagnosis, no specific autoantibody markers or liver biopsy were available to confirm this mechanism. Therefore, the observed abnormalities were interpreted cautiously within the broader context of systemic inflammation and critical illness. Of particular diagnostic significance was the presence of extreme hyper-CK levels CPK >3,000 U/L), markedly elevated CPK-MB (Figure 1), and increased aldolase, reflecting active immune-mediated muscle fiber necrosis and correlating with the patient’s profound muscle weakness and dysphagia.

Autoimmune testing revealed high titers of ANA 1:640, with a fine granular nuclear pattern, positive anti-dsDNA antibodies, and detected anti-Mi2α/β antibodies. These findings strongly supported the diagnosis of an autoimmune overlap syndrome combining dermatomyositis and systemic lupus erythematosus. The ANA pattern was further confirmed by indirect immunofluorescence on HEp-2 cells, demonstrating a fine granular nuclear pattern (ICAP AC-4/AC-5), consistent with antibodies directed against extractable nuclear antigens characteristic of connective tissue diseases.

Electrophysiologic, histopathologic, and imaging correlation

**Figure 2:**
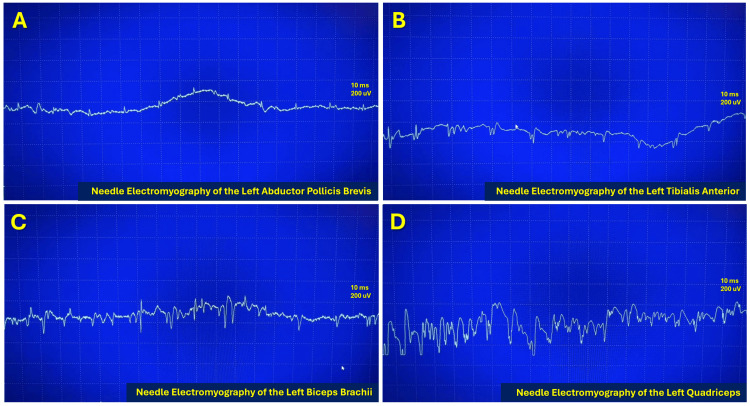
Electrophysiologic findings on needle electromyography obtained from different muscle groups A: Left abductor pollicis brevis; B: Left tibialis anterior; C: Left biceps brachii; D: Left quadriceps All recordings demonstrate abnormal spontaneous activity, reduced recruitment, and low-amplitude, short-duration motor unit potentials. In conjunction with motor nerve conduction studies showing markedly reduced compound muscle action potential amplitudes with preserved conduction velocities and entirely normal sensory nerve conduction studies, these findings support a severe motor axonal polyneuropathy rather than an isolated necrotizing myopathy or primary demyelinating process.

Electrophysiologic studies (Figure 2) demonstrated severe motor axonal polyneuropathy without evidence of demyelination, characterized by markedly reduced compound muscle action potential amplitudes, relatively preserved conduction velocities, and absence of temporal dispersion or conduction block. Importantly, sensory nerve conduction studies were within normal limits, arguing against a generalized sensory-motor demyelinating neuropathy and supporting a predominantly motor axonal process. Needle electromyography performed in representative proximal and distal muscles revealed diffuse abnormal spontaneous activity, reduced recruitment, and low-amplitude, short-duration motor unit potentials. Although such findings may be observed in severe necrotizing myopathies, the concomitant reduction in motor amplitudes on nerve conduction studies supports secondary axonal involvement rather than an isolated myopathic process. The symmetric and multisegmental distribution further excluded focal or compressive etiologies. Collectively, these findings favor immune-mediated neuromuscular injury with overlapping inflammatory myopathy and secondary motor axonal polyneuropathy, helping to exclude classic Guillain-Barré variants and reinforcing the systemic autoimmune nature of the disease process.

**Figure 3:**
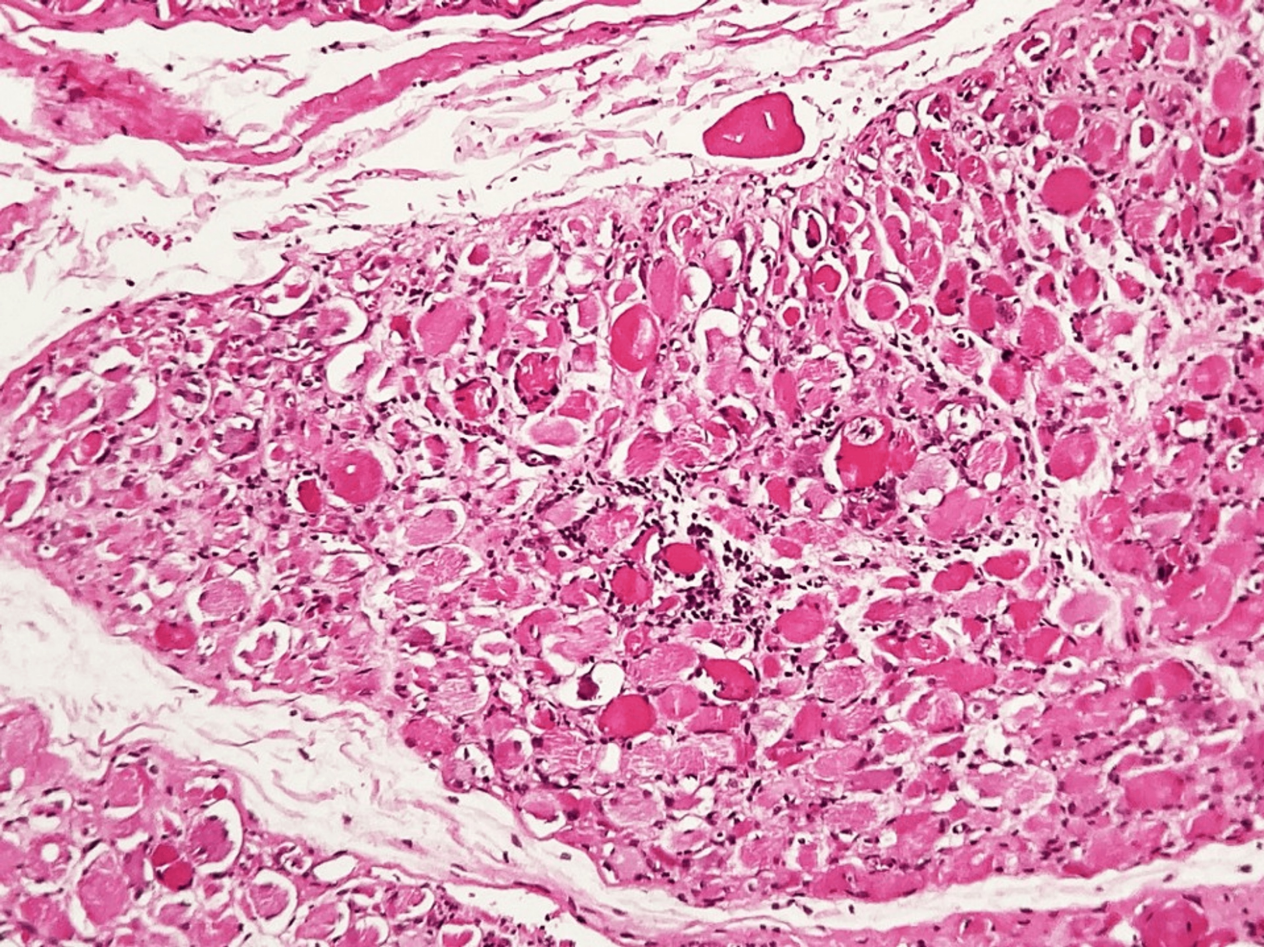
Predominantly perifascicular muscle fiber atrophy associated with an inflammatory infiltrate composed of lymphocytes and macrophages, as well as myocyte necrosis Hematoxylin and eosin stain; bright-field light microscopy. Total magnification ×100.

**Figure 4:**
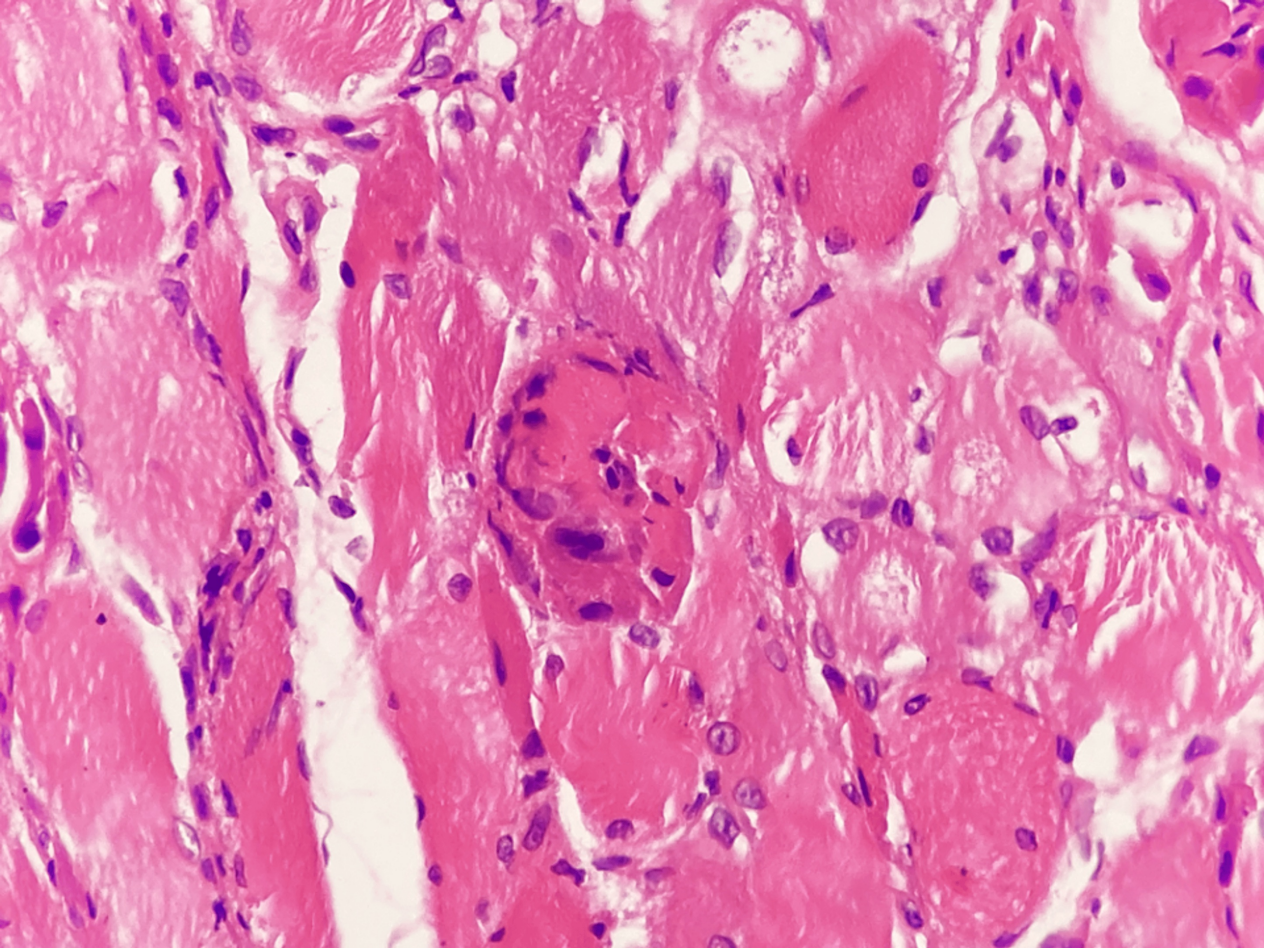
Necrotic myocyte associated with inflammatory infiltrate Hematoxylin and eosin stain; bright-field light microscopy. Total magnification ×400.

To further clarify the etiology of the myopathy, a muscle biopsy of the right vastus lateralis was performed, providing definitive diagnostic confirmation. Histopathologic examination revealed predominant perifascicular muscle fiber atrophy, a hallmark feature of dermatomyositis, associated with inflammatory infiltrates composed of lymphocytes and macrophages, as well as focal myocyte necrosis (Figure 3). These findings reflect immune-mediated microvascular injury and perifascicular ischemic stress. At higher magnification, necrotic myocytes surrounded by inflammatory cells were clearly identified (Figure 4), confirming active muscle fiber destruction rather than a chronic or burnout process.

**Figure 5:**
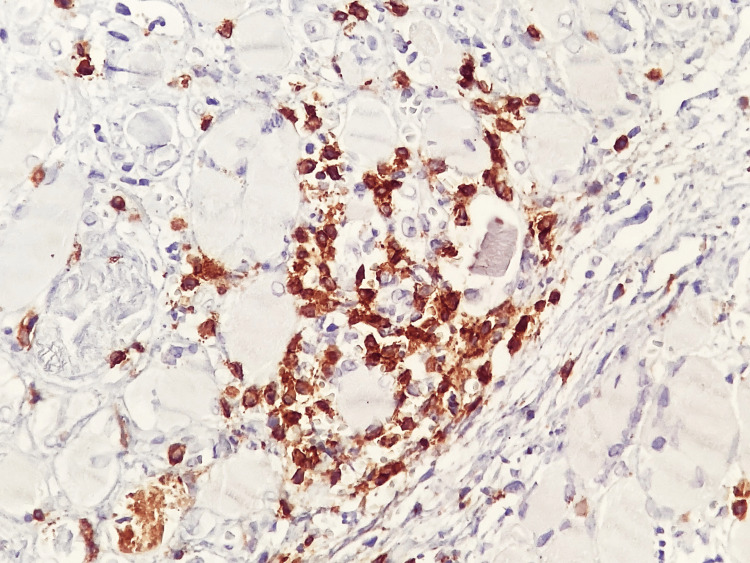
Abundant inflammatory infiltrate of CD4⁺ T lymphocytes between muscle fibers Immunohistochemistry using anti-CD4 antibody; bright-field light microscopy. Total magnification ×400.

**Figure 6:**
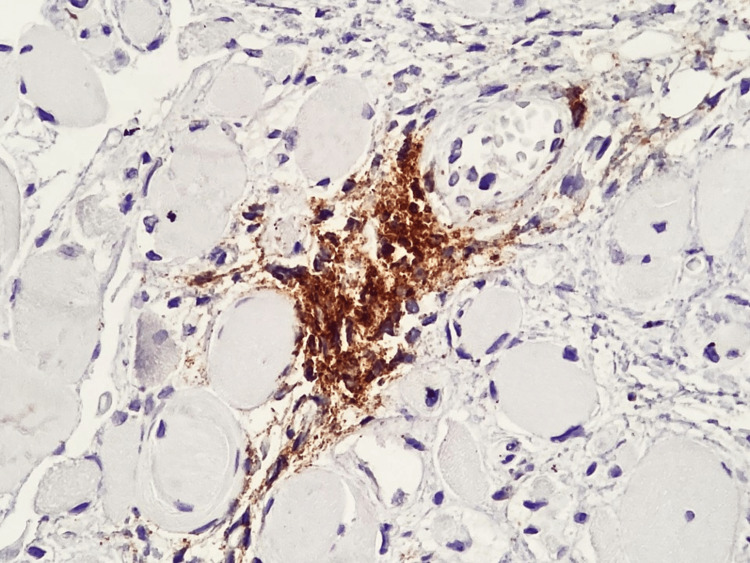
Perivascular-predominant inflammatory infiltrate of CD20⁺ B lymphocytes Immunohistochemistry using anti-CD20 antibody; bright-field light microscopy. Total magnification ×400.

Immunohistochemical analysis allowed further characterization of the inflammatory milieu and provided insight into the underlying immunopathogenesis. Abundant CD4⁺ T lymphocytes were observed between muscle fibers (Figure 5), indicating a prominent cell-mediated immune response consistent with T-cell-driven muscle injury. In parallel, CD20⁺ B lymphocytes showed a predominantly perivascular distribution (Figure 6), supporting the contribution of humoral immunity and autoantibody-mediated mechanisms, which are particularly relevant in dermatomyositis and overlap syndromes with SLE.

**Figure 7:**
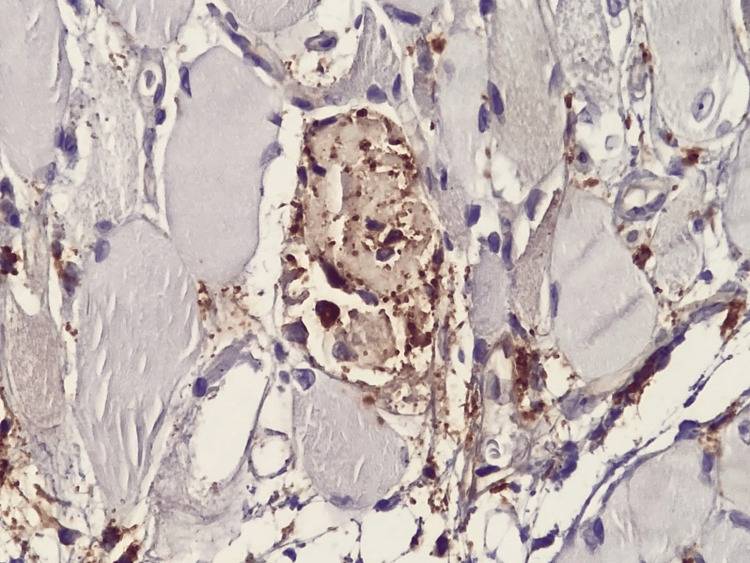
Muscle fiber destruction associated with macrophage inflammatory infiltrate Immunohistochemistry using anti-CD68 antibody; bright-field light microscopy. Total magnification ×400.

**Figure 8:**
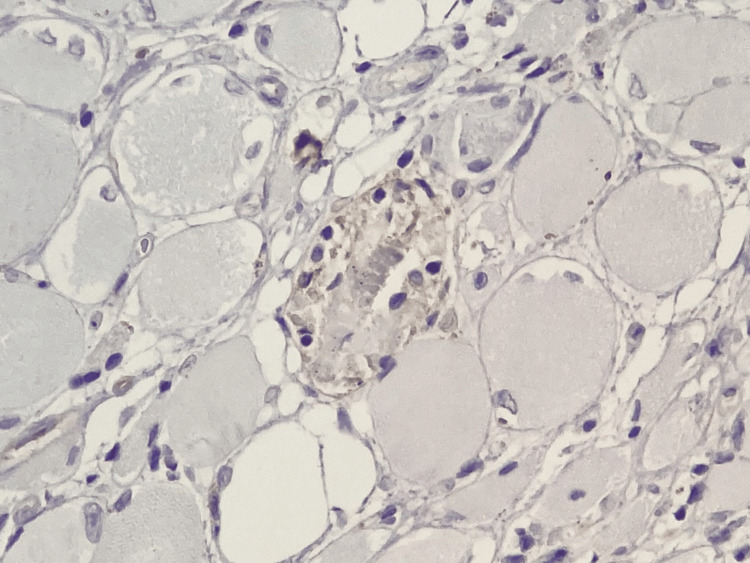
Deposition of anti-MHC class II antibody on a necrotic muscle fiber Immunohistochemistry using anti-MHC class II antibody; bright-field light microscopy. Total magnification ×400.

**Figure 9:**
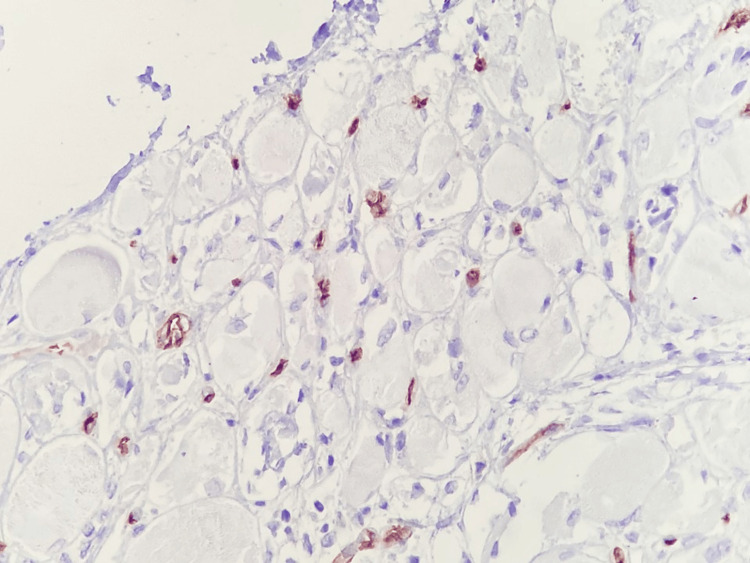
Preserved vascular expression pattern of CD34 within muscle vasculature Immunohistochemistry using anti-CD34 antibody; bright-field light microscopy. Total magnification ×400.

Macrophage involvement was highlighted by strong CD68 positivity, demonstrating active phagocytosis and macrophage-mediated muscle fiber destruction (Figure 7). In addition, deposition of major histocompatibility complex (MHC) class II molecules on necrotic muscle fibers (Figure 8) reflected aberrant antigen presentation at the myocyte level, further supporting immune activation and loss of self-tolerance within skeletal muscle tissue. Notably, CD34 immunostaining demonstrated preserved vascular architecture (Figure 9), thereby excluding advanced ischemic injury or fibrotic remodeling and indicating an inflammatory process that is potentially reversible with appropriate immunomodulatory therapy.

**Figure 10:**
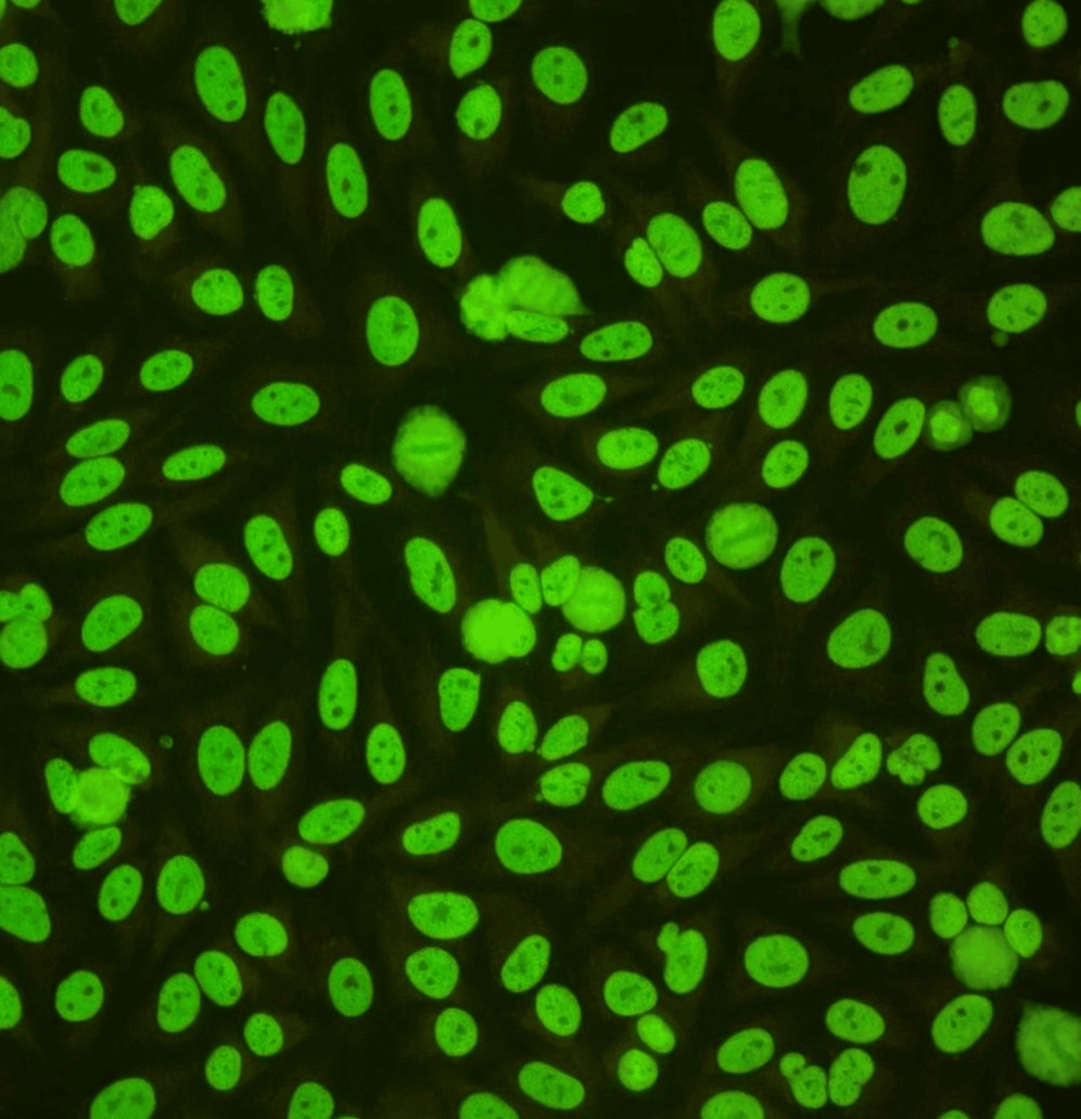
Indirect immunofluorescence microphotograph on HEp-2 cells for ANA determination showing a fine granular nuclear pattern at a titer of 1:640 ANA: Antinuclear antibodies

Immunologic evaluation demonstrated high-titer ANA (1:640). The ANA pattern was further characterized by indirect immunofluorescence on HEp-2 cells, which revealed a fine granular nuclear pattern with diffuse, homogeneous fine speckled nuclear fluorescence, preservation of nucleolar delineation, and absence of cytoplasmic or nuclear envelope staining (Figure 10). This pattern corresponds to ICAP AC-4/AC-5 and is classically associated with autoantibodies directed against extractable nuclear antigens, including anti-Sm, anti-RNP, anti-SSA/Ro, and anti-Mi-2, which are characteristic of systemic autoimmune connective tissue diseases such as SLE and dermatomyositis. In the present case, this immunofluorescence finding was concordant with the simultaneous positivity of ANA 1:640, anti-dsDNA antibodies, and detected anti-Mi2α/β antibodies, confirming a high-serologic-activity autoimmune overlap syndrome consistent with dermatomyositis-lupus.

**Figure 11:**
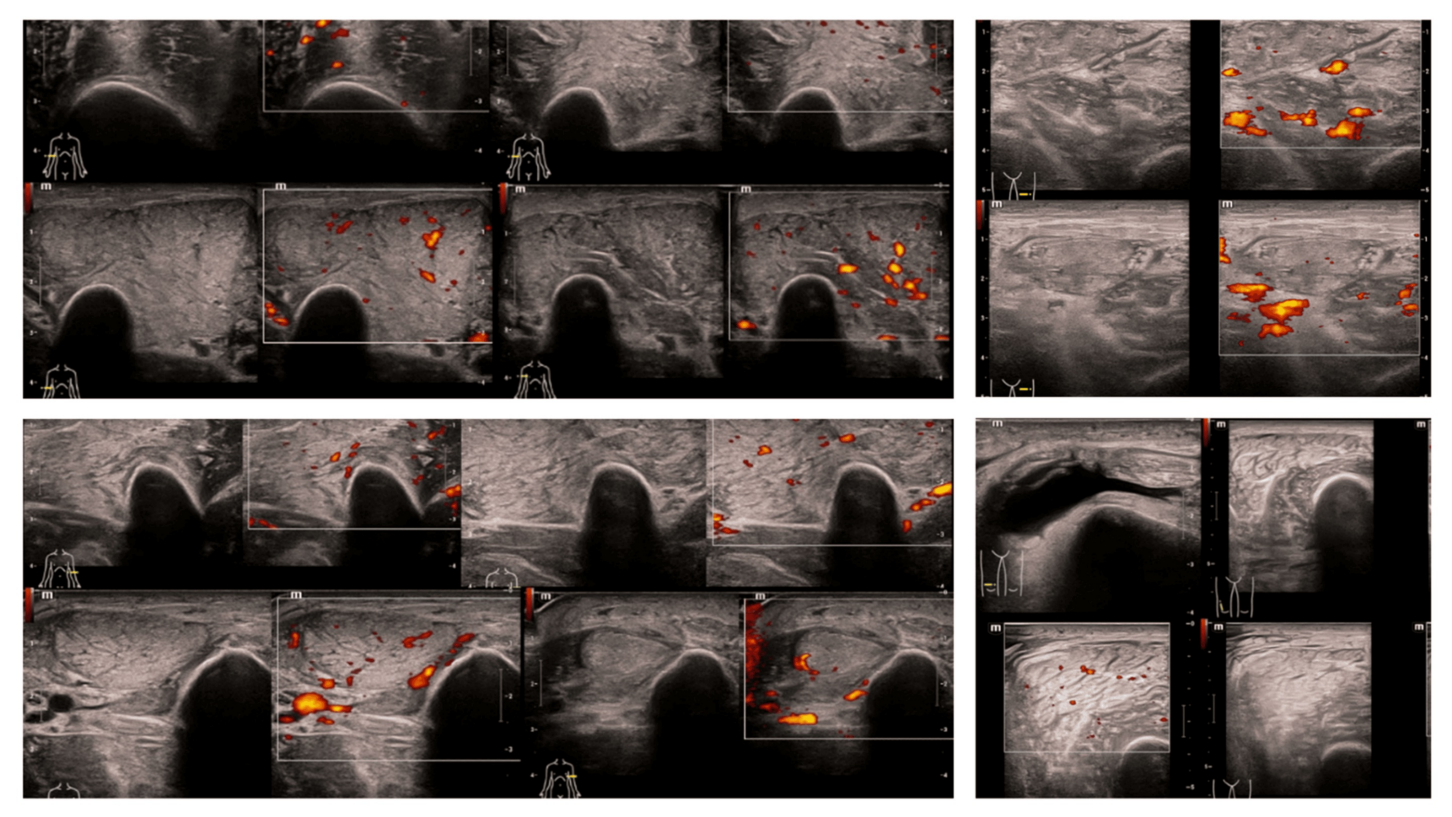
Musculoskeletal ultrasound with color Doppler evaluation of bilateral upper and lower extremity muscle groups A: Transverse view of an upper extremity muscle demonstrating increased muscle thickness and mildly increased echogenicity with scattered intramuscular Doppler signals, indicating active hyperemia; B: Contralateral upper extremity muscle showing a similar pattern of diffuse echogenicity and low-to-moderate intramuscular vascular flow on color Doppler, consistent with symmetric inflammatory involvement; C: Upper extremity muscle with preserved fascial planes but increased intramuscular Doppler activity, suggestive of inflammatory hypervascularity without abscess formation; D: Corresponding contralateral muscle demonstrating patchy vascular signals within thickened muscle fibers, maintaining bilateral symmetry; E: Longitudinal view of upper extremity musculature showing diffuse hypoechoic changes compatible with edema and mild Doppler flow enhancement; F: Similar longitudinal assessment on the opposite side confirming symmetric structural and vascular alterations; G: Lower extremity muscle displaying marked intramuscular Doppler activity with heterogeneous echotexture and increased muscle bulk; H: Contralateral lower extremity muscle with moderate-to-marked hypervascularity and preserved deep fascial contours; I: Lower extremity muscle demonstrating multifocal Doppler signals within edematous muscle fibers, without evidence of fluid collection; J: Longitudinal lower extremity view showing architectural distortion secondary to inflammatory edema, without venous thrombosis; K: Prominent Doppler signal within lower extremity musculature, reflecting active inflammatory hyperemia; L: Bilateral comparison image demonstrating diffuse increased echogenicity and mild vascular activity, consistent with systemic inflammatory myopathy

Complementary imaging with muscle ultrasound of the upper and lower extremities demonstrated bilateral, symmetric, and diffuse muscle involvement, characterized by increased muscle thickness, increased echogenicity, and mild-to-moderate intramuscular vascularity, without evidence of abscess formation or thrombosis (Figure 11). This ultrasonographic pattern was highly suggestive of active inflammatory myopathy and correlated with both the histopathologic findings and the subacute phase of immunologic activity. The absence of advanced fibrosis or focal collections further supported the diagnosis of an active, potentially treatment-responsive inflammatory process. Taken together, the integration of electrophysiologic findings, muscle histopathology, immunohistochemistry, and imaging provided a coherent and comprehensive depiction of immune-mediated muscle injury, strongly supporting the diagnosis of anti-Mi2-positive dermatomyositis with SLE overlap and explaining the severity of the patient’s neuromuscular and respiratory manifestations.

Clinical deterioration and intensive care management

Despite initial immunosuppressive therapy, the patient experienced progressive respiratory compromise, manifested by tachypnea, increased work of breathing, and hypoxemia. She was transferred to the intensive care unit, where orotracheal intubation and invasive mechanical ventilation were initiated. During central venous catheter placement, a right-sided iatrogenic pneumothorax occurred and was promptly managed with a chest tube insertion, with complete resolution.

Given the lack of adequate response to high-dose corticosteroids and the presence of life-threatening neuromuscular and respiratory involvement, IVIG therapy was initiated, resulting in partial clinical improvement. However, persistent muscle weakness, elevated muscle enzymes, and ventilator dependence prompted escalation to therapeutic PLEX as a rescue immunomodulatory strategy.

Therapeutic PLEX and clinical response

**Figure 12:**
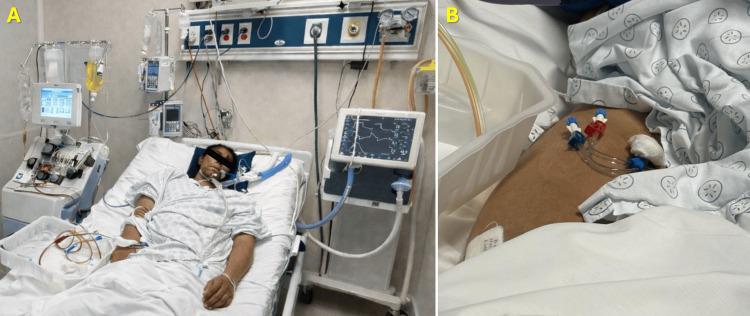
Bedside implementation of therapeutic PLEX in a mechanically ventilated critically ill patient A: Patient undergoing therapeutic PLEX while receiving invasive ventilatory support through a tracheostomy. The apheresis device is positioned adjacent to the bed, with the extracorporeal circuit connected via a central venous catheter. Continuous hemodynamic and respiratory monitoring are visible, reflecting the critical care environment and the need for close physiological surveillance during the procedure. B: A close-up view of the double-lumen central venous catheter (Mahurkar type) inserted in the femoral region, with arterial and venous lumens clearly identified. The catheter serves as vascular access for the extracorporeal circuit, enabling plasma separation and replacement during the exchange session. Together, these images document the technical setup and bedside implementation of therapeutic PLEX in a critically ill patient with severe autoimmune inflammatory myopathy. PLEX: Plasma exchange

**Figure 13:**
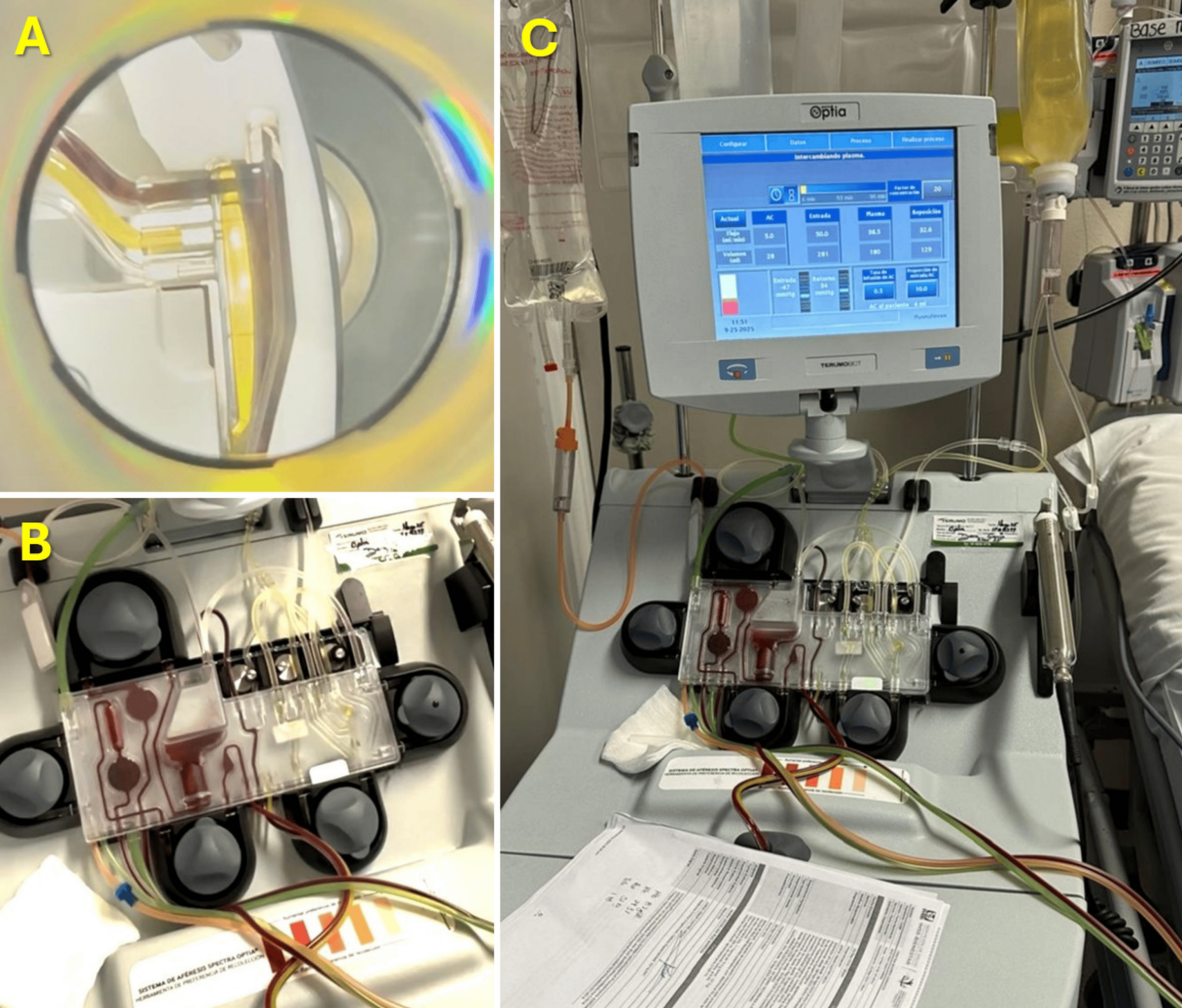
Therapeutic PLEX procedure in the intensive care setting featuring technical components and real-time system monitoring A: A close-up view of the centrifugation chamber during active plasma separation. The distinct yellow plasma layer is clearly visible, separated from the cellular components by centrifugal force, demonstrating effective blood component fractionation. B: The extracorporeal circuit cassette is mounted on the apheresis device, with visible blood flow through the tubing system. The circuit includes the separation chamber, collection reservoirs, and tubing connections, reflecting continuous plasma processing during the exchange session. C: The apheresis platform (Spectra Optia® system) with real-time monitoring parameters visible on the screen, including processed volume and flow metrics. The setup includes replacement fluid (albumin) and continuous hemodynamic monitoring, illustrating a controlled therapeutic PLEX procedure performed in an intensive care setting. PLEX: Plasma exchange

The patient underwent five sessions of therapeutic PLEX, each exchanging 1.5 plasma volumes with albumin as replacement fluid. The procedure was performed via a Mahurkar catheter under continuous hemodynamic monitoring using a Spectra Optia® system (Terumo BCT, Denver, CO, USA), as shown in Figures 12-13. The PLEX was well tolerated, without significant adverse events. After completion of the plasmapheresis regimen, marked clinical and biochemical improvement was observed. Creatine kinase levels decreased by approximately 60%, proximal muscle strength improved to grade 3/5, vasopressor support was discontinued, and respiratory function stabilized, allowing transition to spontaneous ventilation through tracheostomy.

Outcome and follow-up

Although respiratory function improved, the patient continued to experience severe neurogenic dysphagia, requiring placement of a percutaneous endoscopic gastrostomy for definitive nutritional support. Endocrinologic evaluation revealed thyroid dysfunction characterized by elevated thyroid-stimulating hormone (TSH) levels consistent with primary autoimmune hypothyroidism, and thyroid hormone replacement therapy was initiated. Transient alterations in other pituitary axes were interpreted cautiously in the context of critical illness, recognizing the possibility of non-thyroidal illness syndrome and adaptive neuroendocrine responses rather than definitive structural hypopituitarism. A structured rehabilitation program focused on respiratory and motor recovery was implemented.

At the most recent follow-up, the patient remained hemodynamically stable, with preserved renal and hepatic function and gradual improvement in neuromuscular strength. She continues multidisciplinary outpatient follow-up with intensive care medicine, rheumatology, neurology, endocrinology, rehabilitation, and speech therapy. Although the long-term prognosis remains guarded, the observed clinical trajectory suggests a favorable response to combined immunomodulatory therapy.

## Discussion

Idiopathic inflammatory myopathies are heterogeneous autoimmune musculoskeletal diseases, with a global incidence estimated between one and 10 cases per million inhabitants per year [1,2]. Among them, dermatomyositis represents one of the most well-defined entities from a clinical, histopathological, and immunologic standpoint [1,15]. The identification of myositis-specific autoantibodies has allowed the definition of more precise clinical phenotypes and prognostic correlations [3].

In particular, anti-Mi2 antibodies are associated with subacute-onset dermatomyositis, prominent cutaneous involvement, and an excellent response to conventional immunosuppressive therapy, with five-year mortality rates below 10% [4]. However, cases in which dermatomyositis occurs in the context of an overlap syndrome with SLE are uncommon, accounting for approximately 3% to 7% of IIMs, and their recognition poses significant diagnostic and therapeutic challenges [6,13]. Fewer than 60 well-documented cases of dermatomyositis-SLE overlap with both histologic and serologic confirmation have been reported in the literature [6,9].

The coexistence of anti-Mi2, anti-dsDNA, and anti-Ro antibodies is particularly rare [16]. These overlap syndromes are frequently associated with higher inflammatory activity, increased risk of multiorgan involvement, autoimmune liver disease, Raynaud phenomenon, cytopenias, and endocrine abnormalities related to hypothalamic-pituitary-adrenal axis dysfunction [7]. The present case is remarkable due to its severe systemic involvement, extreme elevation of CK levels (>3000 U/L), and the need for multidisciplinary critical care management [9]. Although anti-Mi2-positive patients typically respond favorably to corticosteroids and IVIG, refractory evolution with respiratory compromise prompted the use of therapeutic PLEX as a rescue strategy [1].

Recent studies suggest that sequential administration of IVIG followed by PLEX may accelerate the clearance of circulating autoantibodies and immune complexes, improve muscle strength, and reduce serum CK levels and proinflammatory cytokines [10]. The marked elevation of muscle enzymes observed in this patient likely reflects a highly active immune-mediated necrotizing process [15]. Histopathologic findings of perifascicular necrosis with perivascular lymphocytic infiltrates are consistent with complement-mediated microangiopathy (C5b-9 deposition), a hallmark feature of dermatomyositis. The concomitant presence of positive ANA and anti-dsDNA antibodies, along with hypocomplementemia, supports the diagnosis of a dermatomyositis-SLE overlap syndrome, a recognized but rare entity in which cutaneous and muscular manifestations may overlap with subacute cutaneous lupus or primary lupus myopathy [6].

The initial differential diagnosis included motor axonal variant Guillain-Barré syndrome, given the ascending weakness pattern and albuminocytologic dissociation in CSF. However, electromyography demonstrated diffuse denervation without demyelination, antiganglioside antibodies (GM1, GD1a, and GQ1b) were negative, and muscle biopsy revealed necrosis and inflammation consistent with inflammatory myopathy, allowing this diagnosis to be excluded [8]. Toxic, infectious, and paraneoplastic etiologies were also excluded through appropriate laboratory testing, tumor markers, and protein electrophoresis [8].

Treatment with corticosteroids, IVIG, and five PLEX sessions at 1.5 plasma volumes using albumin replacement resulted in favorable clinical evolution, a 60% reduction in CK levels, and hemodynamic improvement [10]. This outcome is consistent with previous reports demonstrating the efficacy of PLEX in refractory dermatomyositis and acute myopathic crises [14]. Despite the absence of randomized controlled trials, available evidence suggests that early therapeutic PLEX may serve as an effective immunomodulatory bridge, particularly in patients with respiratory failure, severe dysphagia, or rapidly progressive muscle weakness [16,17]. This case underscores the importance of comprehensive immunologic evaluation in patients with severe myopathies and highlights the value of a multidisciplinary approach within the intensive care unit [5]. It also provides a relevant clinical lesson: favorable serologic profiles such as anti-Mi2 positivity do not preclude critical presentations or the coexistence of other major autoimmune diseases. Limitations of this report include the inability to perform spinal and brain MRI due to logistical constraints and the absence of longitudinal functional muscle biomarkers; nevertheless, the clinical and biochemical course supports a strong causal correlation [13].

A key contribution of this case is the demonstration of the diagnostic value of an integrated, multimodal approach. The concordance between high-activity autoimmune serology, electrophysiologic evidence of severe motor axonal involvement without demyelination, characteristic histopathologic findings of active immune-mediated myopathy, and supportive imaging findings allowed accurate identification of the underlying disease process. This comprehensive evaluation was essential to distinguish this condition from other acute neuromuscular disorders and to guide timely therapeutic escalation in a rapidly deteriorating clinical context. In conclusion, this case provides observational evidence that sequential IVIG and therapeutic PLEX may represent a safe and effective strategy in anti-Mi2-positive dermatomyositis with SLE overlap and critical illness, emphasizing the importance of early recognition and timely immunomodulatory intervention in highly complex clinical scenarios [18,19].

## Conclusions

This case describes a rare and severe autoimmune overlap syndrome involving anti-Mi2-positive dermatomyositis and SLE, presenting with rapidly progressive neuromuscular weakness and respiratory failure requiring intensive care management. Although anti-Mi2-associated dermatomyositis is generally considered to carry a favorable prognosis, this report highlights that it may, in uncommon instances, evolve into a life-threatening multisystem condition.

Clinical stabilization and biochemical improvement were observed following sequential multimodal immunomodulatory therapy administered in the intensive care setting. Given the temporal overlap between high-dose corticosteroids, IVIG, and therapeutic PLEX, the observed improvement should be interpreted as a cumulative response to combined immunomodulatory strategies rather than attributable to a single intervention. This case emphasizes the importance of early recognition of severe phenotypes, comprehensive diagnostic integration, and coordinated multidisciplinary management. It reinforces the need for individualized escalation strategies in critically ill patients with inflammatory myopathies and complex autoimmune overlap syndromes.
